# *Plectus* of the Prairie: A Case Study of Taxonomic Resolution from a Nematode Biodiversity Survey

**DOI:** 10.2478/jofnem-2022-0039

**Published:** 2022-10-29

**Authors:** Abigail Borgmeier, Kaitlin Gattoni, Tim Harris, Rebecca Higgins, Peter Mullin, Dorota Porazinska, Kirsten Powers, David Wedin, Thomas Powers

**Affiliations:** 1 Department of Plant Pathology, University of Nebraska-Lincoln, Lincoln, Nebraska; 2 Department of Entomology and Nematology, University of Florida, Gainesville, Florida; 3 School of Natural Resources, University of Nebraska-Lincoln, Lincoln, Nebraska

**Keywords:** COI, DNA barcoding, native plant communities, nematode distribution, tallgrass prairie, taxonomy

## Abstract

Taxonomic resolution is a critical component of biodiversity assessments. In this case study, we examined a single taxon within a larger study of nematode diversity to evaluate the taxonomic resolution of different diversity assessment methods. The selected taxon was the microbial-feeding genus *Plectus*, a group considered to include multiple cosmopolitan species. The methods included a morphological evaluation by light microscopy, Sanger sequencing of PCR amplicons of COI and 18S gene regions, and 18S metabarcoding sequencing. The study sites were 15 remnant tallgrass prairie plots in eastern Nebraska. In the morphological analysis, we observed two basic morphotypes, a short-tailed form with a small amphid and a long-tailed form with a large amphid. Sanger sequencing of COI sorted *Plectus* diversity into six distinct clades. The largest two of these six clades keyed to *P. parietinus* and *P. rhizophilus* based on morphology. BLAST analysis with COI revealed no close matches in GenBank. Sanger sequencing of the 18S region did not differentiate the six clades. These results illustrate that the method of diversity assessment strongly influences estimates of biodiversity. An additional 95 *Plectus* specimens, from outside the remnant sites, added taxonomic breadth to the COI phylogenetic tree. There were no geographically widespread COI haplotypes and no evidence of cosmopolitan *Plectus* species.

Current surveys of nematode diversity frequently apply DNA barcoding or metabarcoding as a method of assessment (Floyd et al., 2002; Treonis et al., 2018; Schenk and Fontaneto, 2020). The objective of the survey generally dictates the assessment method of choice. If the objective is to assess functional diversity, e.g., trophic groups, molecular methods may not be required and traditional morphological evaluation may be adequate (Hodda and Wanless, 1994; Bloemers et al., 1997). If taxon/species diversity is a goal, then a method of greater taxonomic resolution, such as DNA barcoding, may be necessary. And, if the scope of the survey requires multiple comparisons across broad geographic regions and scales, then a metabarcoding approach may be optimal (Porter and Hajibabaei, 2018; Brunbjerg et al., 2019; Arribas et al., 2021). Each of these methods has advantages and disadvantages, but one common feature of the molecular methods is the need for referral to a DNA database. In this study we chose a single taxon, *Plectus* Bastian, 1865, and used three standard methods of diversity assessment, to determine the extent to which method and database composition influence taxonomic resolution and the selection of reportable taxonomic units.

*Plectus* species were examined from 15 remnant (never plowed) tallgrass prairie sites outside of Lincoln, Nebraska. These 15 sites, located within a larger restoration project called the Lancaster County Prairie Corridor, were designed to maximize habitat continuity. The sites have been variously managed by burning, grazing, or mowing, resulting in communities of different species compositions representing a range of vegetation “quality” with regard to native tallgrass prairie plants (Chamberlain and Ingram, 2012). In the terrestrial nematode survey, *Plectus* stood out as a taxon present in all samples, across the variety of management regimes, and relatively abundant in each of the sampled sites. *Plectus* is considered a cosmopolitan genus, found in soils and freshwater habitats, and many *Plectus* species are reported to have a worldwide distribution (Overgaard, 1967; de Goede and Bongers, 1994; Sohlenius et al., 1996; Holovachov, 2004; Iliev and Ilieva, 2016; Naumova et al., 2017). In the first major study of nematode diversity in North American grasslands, Orr and Dickerson (1966) identified six *Plectus* species from the Flint Hills of Kansas, the present-day location of the Konza Prairie Long-Term Ecological Reserve (Knapp, 1998). These included *P. acuminatus* Bastian, 1865, *P. armatus* Butschli, 1873, *P. assimilis* Butschli, 1873, *P. parietinus* Bastian, 1865, *P. rhizophilus* de Mann, 1880, and *P. varians* Maggenti, 1961.

Most known *Plectus* species are parthenogenetic, while some readily undergo anhydrobiosis, and can be recovered from extreme habitats including Antarctic dry valleys (Kagoshima et al., 2012; Adams et al., 2014), arctic tundra, and high alpine (Porazinska et al., 2021). They are not known from marine sediments. There are 76 species in the genus (Schmidt-Rhaesa et al., 2013). In GenBank, there are 22 *Plectus* species represented with a Linnaean binomial, and 57 *Plectus* DNA sequences that lack an assigned species name. In SILVA, the ribosomal RNA database, there are 14 *Plectus* species with a Linnaean binomial and approximately 20 isolates that lack an assigned species name.

There were two objectives for this *Plectus* case study.

1. To determine the level of taxonomic resolution provided by each assessment method as it applies to *Plectus* specimens collected from the 15 remnant Prairie Corridor sites. The three assessment methods included a traditional morphological approach, barcoding using 18S and COI genetic markers, and metabarcoding using 18S.
2. To molecularly compare *Plectus* diversity and identity of Prairie Corridor specimens, with GenBank accessions and specimens collected by the authors from other grassland and non-grassland habitats across a broad geographic scale.

## Materials and Methods

### Soil collection and nematode isolation

**Figure 1:**
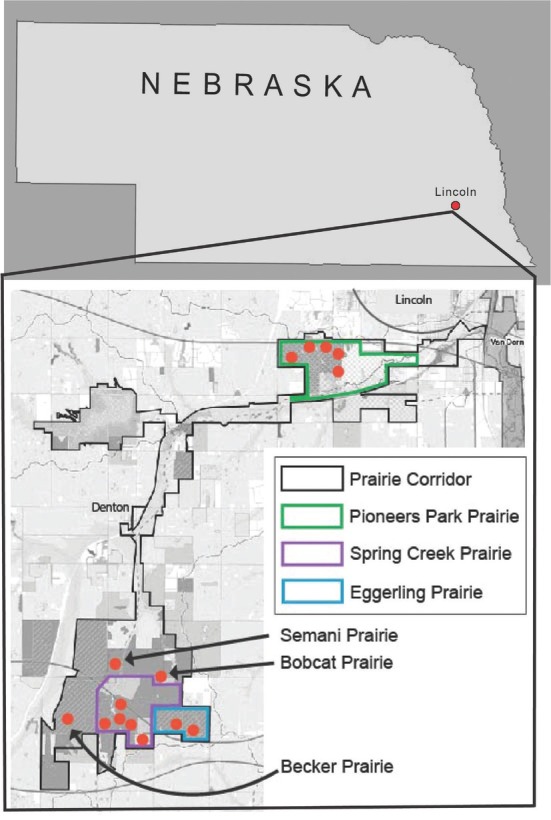
Map of Nebraska and location of the 16-km-long Lancaster County Prairie Corridor. Orange dots indicate the 15 remnant prairie sites.

Fifteen sites were sampled within the 16-km-long Prairie Corridor (Fig. 1). The 15 sites were selected because, as remnant prairies, they had never been plowed or converted to agricultural production. Each site ranged from 0.8 ha to 1.2 ha in size, and soil cores were taken from a 40 m × 40 m square in the center of each site following a protocol to ensure standardized soil sampling at each location (Neher et al., 1995). Soil cores were taken at regular intervals throughout the 40 m × 40 m grid at a depth of approximately 20 cm using an Oakfield Soil Corer (Oakfield Apparatus, Oakfield, Wisconsin) with a 2.5-cm diameter. All soil cores from a single 40 m × 40 m square were bulked and stored at 8°C prior to nematode extraction.

### Morphological approach

Nematodes were extracted from 200 cc of soil via the sieving and sugar centrifugation method (Jenkins, 1964). Nematodes were counted, and a total of 150 nematodes was identified to genus using morphological characters. Twenty-five of the 150 nematodes were photographed at ×200 and ×400 magnifications to serve as photographic vouchers, and immediately processed for DNA barcoding to preserve the linkage between DNA and morphology. Voucher images were taken of the full body, head, and tail with a Leica DC300 video camera (leica-microsystems.com) mounted on a Leica DMLB light microscope with differential interference contrast. The taxonomic keys and compendia of Andrassy (1985), Bongers (1989), Ebsary (1985), Holovachov and De Ley (2006), Holovachov (2014), and Maggenti (1961) were used for a morphology-based identification of *Plectus* species. DNA was extracted from the photographed specimens by rupturing the nematode in an 18-mL droplet of sterilized water, which was stored at -22°C until PCR (Powers et al., 2014). *Plectus* specimens from additional sites (a total of 95 specimens from 28 sites) including two well-studied tallgrass prairies, Konza and Nine-Mile Prairies, were added to expand taxonomic breadth and facilitate comparisons across broader geographic regions and ecosystems (Table 1).

**Table 1:** Specimen collection information.

Group	NID	Species	Site	Location	Marker	GB Accession #
1	N11040	*Plectus frigophilus*	Lake Bonney	McMurdo Dry Valleys, Antarctica	COI	OP208981
1	N11213	*Plectus frigophilus*	Lake Fryxell	McMurdo Dry Valleys, Antarctica	COI	OP208968
1	N11461	*Plectus frigophilus*	Lake Fryxell	McMurdo Dry Valleys, Antarctica	COI	OP208969
1	N11211	*Plectus frigophilus*	Lake Fryxell	McMurdo Dry Valleys, Antarctica	COI	OP208970
1	N11110	*Plectus frigophilus*	Lake Fryxell	McMurdo Dry Valleys, Antarctica	COI	OP208971
1	N11042	*Plectus frigophilus*	Lake Bonney	McMurdo Dry Valleys, Antarctica	COI	OP208973
1	N11146	*Plectus frigophilus*	Lake Bonney	McMurdo Dry Valleys, Antarctica	COI	OP208976
1	N11041	*Plectus frigophilus*	Lake Bonney	McMurdo Dry Valleys, Antarctica	COI	OP208977
1	N11526	*Plectus frigophilus*	Lake Fryxell	McMurdo Dry Valleys, Antarctica	COI	OP208978
1	N11142	*Plectus frigophilus*	Lake Bonney	McMurdo Dry Valleys, Antarctica	COI	OP208979
1	N11215	*Plectus frigophilus*	Lake Fryxell	McMurdo Dry Valleys, Antarctica	COI	OP208980
1	N11161	*Plectus frigophilus*	Lake Bonney	McMurdo Dry Valleys, Antarctica	COI	OP208982
1	N11035	*Plectus frigophilus*	Lake Bonney	McMurdo Dry Valleys, Antarctica	COI	OP208983
1	N11034	*Plectus frigophilus*	Lake Bonney	McMurdo Dry Valleys, Antarctica	COI	OP208984
1	N11109	*Plectus frigophilus*	Lake Fryxell	McMurdo Dry Valleys, Antarctica	COI	OP208985
1	N11121	*Plectus frigophilus*	Lake Fryxell	McMurdo Dry Valleys, Antarctica	COI	OP208986
1	N11140	*Plectus frigophilus*	Lake Bonney	McMurdo Dry Valleys, Antarctica	COI	OP208987
1	N11216	*Plectus frigophilus*	Lake Fryxell	McMurdo Dry Valleys, Antarctica	COI	OP208989
1	N11227	*Plectus frigophilus*	Lake Fryxell	McMurdo Dry Valleys, Antarctica	COI	OP208990
1	N11218	*Plectus frigophilus*	Lake Fryxell	McMurdo Dry Valleys, Antarctica	COI	OP208992
1	N11473	*Plectus frigophilus*	Lake Fryxell	McMurdo Dry Valleys, Antarctica	COI	OP208993
1	N11209	*Plectus frigophilus*	Lake Fryxell	McMurdo Dry Valleys, Antarctica	COI	OP208988
1	N12079	*Plectus frigophilus*	Lake Bonney	McMurdo Dry Valleys, Antarctica	COI	OP208991
1	N11107	*Plectus frigophilus*	Lake Fryxell	McMurdo Dry Valleys, Antarctica	COI	OP208972
1	N11033	*Plectus frigophilus*	Lake Bonney	McMurdo Dry Valleys, Antarctica	COI	OP208974
1	N11047	*Plectus frigophilus*	Garwood Valley	McMurdo Dry Valleys, Antarctica	COI	OP208975
2	N11873	*Plectus* sp. 2	Barley field	Hall County, MT	COI	OP208994
2	N11874	*Plectus* sp. 2	Barley field	Hall County, MT	COI	OP208995
3	N13530	*Plectus* sp. 3	Spring Creek Prairie	Prairie Corridor, Lancaster County, NE	COI	OP209006
3	N13357	*Plectus* sp. 3	Eggerling Prairie	Prairie Corridor, Lancaster County, NE	COI	OP209005
3	N13255	*Plectus* sp. 3	Spring Creek Prairie	Prairie Corridor, Lancaster County, NE	COI	OP209004
3	N13045	*Plectus* sp. 3	Pioneers Park Prairie	Prairie Corridor, Lancaster County, NE	COI	OP209003
3	N11765	*Plectus* sp. 3	Spring Creek Prairie	Prairie Corridor, Lancaster County, NE	COI	OP209001
3	N13543	*Plectus* sp. 3	Becker Prairie	Prairie Corridor, Lancaster County, NE	COI	OP209000
3	N11764	*Plectus* sp. 3	Spring Creek Prairie	Prairie Corridor, Lancaster County, NE	COI	OP209002
3	N13030	*Plectus* sp. 3	Semani Prairie	Prairie Corridor, Lancaster County, NE	COI	OP208999
3	N12485	*Plectus* sp. 3	Eggerling Soybean Field	Prairie Corridor, Lancaster County, NE	COI	OP208998
3	N13239	*Plectus* sp. 3	Pioneers Park Prairie	Prairie Corridor, Lancaster County, NE	COI 18S	OP208996 OP205456
3	P194018	*Plectus* sp. 3	Konza Prairie	Riley County, KS	COI	OP208997
a	N11865	*Plectus* sp.	Arctic Preserve	Utqiaghk, AK	COI	OP209007
4	N13608	*Plectus* sp. 4	Eggerling Prairie	Prairie Corridor, Lancaster County, NE	COI	OP209009
4	N13280	*Plectus* sp. 4	Pioneers Park Prairie	Prairie Corridor, Lancaster County, NE	COI 18S	OP209008 OP205457
5	KU759331.1	*Plectus parietinus*	GenBank		COI	KU759331.1
5	KU759327.1	*Plectus parietinus*	GenBank		COI	KU759327.1
5	KU759330.1	*Plectus parietinus*	GenBank		COI	KU759330.1
6	N13176	*Plectus* sp. 6	Eggerling Prairie	Prairie Corridor, Lancaster County, NE	COI 18S	OP209010 OP205453
6	N13287	*Plectus* sp. 6	Spring Creek Prairie	Prairie Corridor, Lancaster County, NE	COI	OP209011
6	N13425	*Plectus* sp. 6	Spring Creek Prairie	Prairie Corridor, Lancaster County, NE	COI	OP209012
6	N13614	*Plectus* sp. 6	Eggerling Prairie	Prairie Corridor, Lancaster County, NE	COI	OP209013
a	N8809	*Plectus* sp.	Brendan C. Byrne State Forest	Burlington County, NJ	COI	OP209014
7	N1023	*Plectus* sp. 7	LeConte ATBI Site	Great Smoky Mountains Nat’l Park	COI	OP209016
7	N4342	*Plectus* sp. 7	Brushy Mountain ATBI Site	Great Smoky Mountains Nat’l Park	COI	OP209015
8	N8808	*Plectus* sp. 8	Brendan C. Byrne State Forest	Burlington County, NJ	COI	OP209017
8	N8817	*Plectus* sp. 8	Brendan C. Byrne State Forest	Burlington County, NJ	COI	OP209018
8	N8828	*Plectus* sp. 8	Brendan C. Byrne State Forest	Burlington County, NJ	COI	OP209019
9	N4244	*Plectus* sp. 9	Canyonlands South	Big Thicket National Preserve, TX	COI	OP209020
9	N9213	*Plectus* sp. 9	Maddron Bald	Great Smoky Mountains Nat’l Park	COI	OP209021
9	N9237	*Plectus* sp. 9	Maddron Bald	Great Smoky Mountains Nat’l Park	COI	OP209022
9	N8797	*Plectus* sp. 9	Brendan C. Byrne State Forest	Burlington County, NJ	COI	OP209023
10	N8706	*Plectus* sp. 10	Lesund	Møre og Romsdal, Norway	COI	OP209024
a	N9166	*Plectus* sp.	Brendan C. Byrne State Forest	Burlington County, NJ	COI	OP209025
11	P87048	*Plectus* sp. 11	Haughton Crater	Devon Island, Canada	COI	OP209026
11	P88010	*Plectus* sp. 11	Haughton Crater	Devon Island, Canada	COI	OP209027
a	P89090	*Plectus* sp.	Nine-Mile Prairie	Prairie Corridor, Lancaster County, NE	COI	OP209028
a	P86098	*Plectus* sp.	Konza Prairie	Riley County, KS	COI	OP209029
a	P89089	*Plectus* sp.	Nine-Mile Prairie	Prairie Corridor, Lancaster County, NE	COI	OP209030
12	KU759349.1	*Plectus parvus*	GenBank		COI	KU759349.1
12	KU759350.1	*Plectus parvus*	GenBank		COI	KU759350.1
12	KU759348.1	*Plectus parvus*	GenBank		COI	KU759348.1
13	N11013	*Plectus murrayi*	Lake Hoare	McMurdo Dry Valleys, Antarctica	COI	OP209035
13	N11018	*Plectus murrayi*.	Lake Fryxell	McMurdo Dry Valleys, Antarctica	COI	OP209036
13	N11028	*Plectus murrayi*	Lake Fryxell	McMurdo Dry Valleys, Antarctica	COI	OP209037
13	N11185	*Plectus murrayi*	Taylor Valley	McMurdo Dry Valleys, Antarctica	COI	OP209038
13	N11199	*Plectus murrayi*	Scott Base	Botany Bay, Antarctica	COI	OP209039
13	N11719	*Plectus murrayi*	Lake Miers Valley	McMurdo Dry Valleys, Antarctica	COI	OP209040
13	N9870	*Plectus murrayi*	Scott Base	Botany Bay, Antarctica	COI	OP209031
13	N9873	*Plectus murrayi*	Scott Base	Botany Bay, Antarctica	COI	OP209032
13	N9880	*Plectus murrayi*	Taylor Valley	McMurdo Dry Valleys, Antarctica	COI	OP209033
13	N9892	*Plectus murrayi*	Hjorth Hill	McMurdo Dry Valleys, Antarctica	COI	OP209034
a	N9135	*Plectus* sp.	Tipple Trail	Medicine Bow-Routt Nat’l Forest, WY	COI	OP209041
14	N8916	*Plectus* sp. 14	Snowy Range	Medicine Bow-Routt Nat’l Forest, WY	COI	OP209042
14	N8869	*Plectus* sp. 14	Happy Jack Trail	Medicine Bow-Routt Nat’l Forest, WY	COI	OP209043
14	N8874	*Plectus* sp. 14	Happy Jack Trail	Medicine Bow-Routt Nat’l Forest, WY	COI	OP209044
15	N12416	*Plectus* sp. 15	Island Lake	Nebraska Sandhills, Garden County, NE	COI	OP209045
15	N12419	*Plectus* sp. 15	Island Lake	Nebraska Sandhills, Garden County, NE	COI	OP209046
15	N12414	*Plectus* sp. 15	Island Lake	Nebraska Sandhills, Garden County, NE	COI	OP209047
15	N12402	*Plectus* sp. 15	Island Lake	Nebraska Sandhills, Garden County, NE	COI	OP209048
16	N12101	*Plectus* sp. 16	Border Lake	Nebraska Sandhills, Garden County, NE	COI	OP209049
16	N12128	*Plectus* sp. 16	Gimlet Lake	Nebraska Sandhills, Garden County, NE	COI	OP209050
16	N12228	*Plectus* sp. 16	Bean Lake	Nebraska Sandhills, Garden County, NE	COI	OP209051
a	N8576	*Plectus* sp.	El Yunque National Forest	Puerto Rico	COI	OP209052
a	N8582	*Plectus* sp.	El Yunque National Forest	Puerto Rico	COI	OP209053
a	N9089	*Plectus* sp.	Henning Conservation Area	Taney County, MO	COI	OP209054
a	P30012	*Plectus* sp.	Konza Prairie	Riley County, KS	COI	OP209055
17	N13180	*Plectus* sp. 17	Eggerling Prairie	Prairie Corridor, Lancaster County, NE	COI 18S	OP209056 OP205467
17	N13460	*Plectus* sp. 17	Eggerling Prairie	Prairie Corridor, Lancaster County, NE	COI	OP209057
a	N11872	*Plectus* sp.	Arctic Preserve	Utqiaghk, AK	COI	OP209058
a	N8374	*Plectus* sp.	Dombas/Hjelle Seter	Oppland, Norway	COI	OP209060
a	N9370	*Plectus* sp.	Happy Jack Trail	Medicine Bow-Routt Nat’l Forest, WY	COI	OP209059
a	N8689	*Plectus* sp.	Hjerkinn	Oppland, Norway	COI	OP209061
a	N9059	*Plectus* sp.	Łukęcin	West Pomerania, Poland	COI	OP209062
a	N11847	*Plectus* sp.	Arctic Preserve	Utqiaghk, AK	COI	OP209063
18	P89028	*Plectus* sp. 18	Konza Prairie	Riley County, KS	COI	OP209066
18	P89030	*Plectus* sp. 18	Konza Prairie	Riley County, KS	COI	OP209064
18	N13465	*Plectus* sp. 18	Eggerling Prairie	Prairie Corridor, Lancaster County, NE	COI	OP209065
18	P117056	*Plectus* sp. 18	Konza Prairie	Riley County, KS	COI	OP209067
19	N9339	*Plectus* sp. 19	Drury-Mincy Conserv. Area	Taney County, MO	COI	OP209068
19	N12733	*Plectus* sp. 19	Twin Creek Prairie	Prairie Corridor, Lancaster County, NE	COI	OP209069
19	P89081	*Plectus* sp. 19	Konza Prairie	Riley County, KS	COI	OP209070
20	P90058	*Plectus* sp. 20	Nine-Mile Prairie	Prairie Corridor, Lancaster County, NE	COI	OP209071
20	P90062	*Plectus* sp. 20	Nine-Mile Prairie	Prairie Corridor, Lancaster County, NE	COI	OP209072
20	P90057	*Plectus* sp. 20	Nine-Mile Prairie	Prairie Corridor, Lancaster County, NE	COI	OP209073
20	N13403	*Plectus* sp. 20	Pioneers Park Prairie	Prairie Corridor, Lancaster County, NE	COI	OP209074
20	N13479	*Plectus* sp. 20	Pioneers Park Prairie	Prairie Corridor, Lancaster County, NE	COI	OP209075
20	N13486	*Plectus* sp. 20	Pioneers Park Prairie	Prairie Corridor, Lancaster County, NE	COI	OP209076
20	N13444	*Plectus* sp. 20	Spring Creek Prairie	Prairie Corridor, Lancaster County, NE	COI	OP209077
20	N13440	*Plectus* sp. 20	Spring Creek Prairie	Prairie Corridor, Lancaster County, NE	COI	OP209078
20	N13329	*Plectus* sp. 20	Pioneers Park Prairie	Prairie Corridor, Lancaster County, NE	COI 18S	OP209083 OP205458
20	N13344	*Plectus* sp. 20	Spring Creek Prairie	Prairie Corridor, Lancaster County, NE	COI 18S	OP209084 OP205459
20	N13225	*Plectus* sp. 20	Pioneers Park Prairie	Prairie Corridor, Lancaster County, NE	COI 18S	OP209082 OP205455
20	N13203	*Plectus* sp. 20	Eggerling Prairie	Prairie Corridor, Lancaster County, NE	COI 18S	OP209081 OP205454
20	N13133	*Plectus* sp. 20	Pioneers Park Prairie	Prairie Corridor, Lancaster County, NE	COI	OP209080
20	N13118	*Plectus* sp. 20	Pioneers Park Prairie	Prairie Corridor, Lancaster County, NE	COI 18S	OP209079 OP205452
20	N13369	*Plectus* sp. 20	Eggerling Prairie	Prairie Corridor, Lancaster County, NE	COI 18S	OP209085 OP205460
20	N13471	*Plectus* sp. 20	Pioneers Park Prairie	Prairie Corridor, Lancaster County, NE	COI	OP209086
20	N13491	*Plectus* sp. 20	Pioneers Park Prairie	Prairie Corridor, Lancaster County, NE	COI	OP209087
20	N13587	*Plectus* sp. 20	Spring Creek Prairie	Prairie Corridor, Lancaster County, NE	COI	OP209088
20	N13598	*Plectus* sp. 20	Spring Creek Prairie	Prairie Corridor, Lancaster County, NE	COI	OP209089
20	N13601	*Plectus* sp. 20	Spring Creek Prairie	Prairie Corridor, Lancaster County, NE	COI	OP209090
20	N13622	*Plectus* sp. 20	Pioneers Park Prairie	Prairie Corridor, Lancaster County, NE	COI	OP209091
20	N13628	*Plectus* sp. 20	Pioneers Park Prairie	Prairie Corridor, Lancaster County, NE	COI	OP209092
20	N13638	*Plectus* sp. 20	Pioneers Park Prairie	Prairie Corridor, Lancaster County, NE	COI	OP209093
20	N13641	*Plectus* sp. 20	Pioneers Park Prairie	Prairie Corridor, Lancaster County, NE	COI	OP209094
20	P86052	*Plectus* sp. 20	Nine-Mile Prairie	Prairie Corridor, Lancaster County, NE	COI	OP209095
20	P89091	*Plectus* sp. 20	Nine-Mile Prairie	Prairie Corridor, Lancaster County, NE	COI	OP209096
20	P90059	*Plectus* sp. 20	Nine-Mile Prairie	Prairie Corridor, Lancaster County, NE	COI	OP209097
20	N12697	*Plectus* sp. 20	Honvlez Prairie	Prairie Corridor, Lancaster County, NE	COI	OP209098
a	P89023	*Plectus* sp.	Haughton Crater	Devon Island, Canada	COI	OP209099
a	P87033	*Plectus* sp.	Konza Prairie	Riley County, KS	COI	OP209100
a	N12491	*Plectus* sp.	Eggerling Soybean Field	Prairie Corridor, Lancaster County, NE	COI	OP209101
a	N12114	*Plectus* sp.	Border Lake	Nebraska Sandhills, Garden County, NE	COI 18S	OP209102 OP205470
a	N13101	*Plectus* sp.	Pioneers Park Prairie	Prairie Corridor, Lancaster County, NE	COI 18S	OP209103 OP205471
a	N12159	*Plectus* sp.	Kokjohn Lake	Nebraska Sandhills, Garden County, NE	COI	OP209104
a	N9790	*Plectus* sp.	Formerly cultivated land	Volcanoes Nat’l Park, Rwanda	COI	OP209105
a	P195005	*Plectus* sp.	Konza Prairie	Riley County, KS	COI	OP209106
	N13211	*Plectus* sp.	Pioneers Park Prairie	Prairie Corridor, Lancaster County, NE	18S	OP205461
	N13250	*Plectus* sp.	Spring Creek Prairie	Prairie Corridor, Lancaster County, NE	18S	OP205462
	N13370	*Wilsonema* sp.	Eggerling Prairie	Prairie Corridor, Lancaster County, NE	18S	OP205469
	P133013	*Anaplectus* sp.	La Selva Biological Station	Costa Rica	18S	OP205466
	P134037	*Plectus* sp.	La Selva Biological Station	Costa Rica	18S	OP205463
	P137018	*Plectus aquatilis*	La Selva Biological Station	Costa Rica	18S	OP205450
	P140029	*Plectus aquatilis*	La Selva Biological Station	Costa Rica	18S	OP205451
	P151063	*Wilsonema* sp.	La Selva Biological Station	Costa Rica	18S	OP205468
	P183015	*Plectus* sp.	Heredia Province	Costa Rica	18S	OP205464
	P183018	*Plectus* sp.	Heredia Province	Costa Rica	18S	OP205465

a Specimen not assigned to a group.

### DNA barcoding

The 25 selected nematode specimens from each site were DNA barcoded targeting specific DNA regions of two different genetic loci, the COI mitochondrial protein coding gene and the 18S ribosomal DNA. The COI primers used were JB3 (5¢-TTTTTTGGGCATCCTGAGGTTTAT-3¢) (Bowles et al., 1992) and JB5 (5¢-AGCACCTAAACTTAAAACATAATGAAAATG-3¢) (Derycke et al., 2005), which produce a 393-bp product once primers are trimmed. PCR was conducted in 0.5-mL thin-wall microcentrifuge tubes containing 30 mL of total volume consisting of 9 mL of the ruptured nematode template, 1.2 mL of double distilled water, 2.4 mL of the forward primer, 2.4 mL of the reverse primer, and 15 mL of JumpStart RED Taq ReadyMix (Sigma-Aldrich, Inc. St. Louis, Missouri, U. S. A.) at a 0.05 U/mL final enzyme concentration. The initial hot start at 94°C for 5 min was followed by 35 cycles of 30 sec of denaturation at 94°C, annealing at 50°C for 30 sec, and extension at 72°C for 90 sec. The final extension occurred once at 72°C for 5 min. Successful PCR products were extracted prior to DNA sequencing from a 7% 1X TAE agarose gel, cleaned using Gel/PCR DNA Fragments Extraction Kit (IBI Scientific, Dubuque, Iowa, U.S.A.), and sent to Eton BioSciences for sequencing in both directions.

The 18S primers were 18s1.2a (5¢-CGATCAGATACCGCCCTAG-3¢) and 18sr2b (5¢-TACAAAGGGCAGGGACGTAAT-3¢), which produce a 593-bp product once primers are trimmed. 18s1.2a is the slightly re-designed 18s1.2 primer that was originally designed using consensus arthropod sequences (Mullin et al., 2003), while 18sr2b is the somewhat redesigned reverse complement of primer rDNA2 from Vrain et al. (1992). This primer set amplifies approximately 630 bp of the 3¢ portion of the 18S ribosomal DNA. PCR amplification of 5 mL of ruptured nematode template was conducted using the same conditions as the COI genetic marker, and 18S amplicon verification, cleaning, and sequencing was as described above.

### Metabarcoding

Nematodes were extracted from a separate 100-cc subsample using soil via sieving and sugar centrifugation method (Jenkins, 1964). Once extracted, all nematodes in each sample were counted under an inverted microscope. Counted nematodes were reduced to 0.5-mL volume, transferred to bead-beating tubes of the PowerSoil DNA Isolation kit (Thermo Fisher Scientific, Waltham, Massachusetts, U.S.A.), and processed according to the manufacturer’s instructions. Extracted DNA of nematodes was processed for amplicon sequencing using the V6–V8 region with NF1-18sr2b primers (Porazinska et al., 2009) producing a 360-bp product using the same PCR protocols. PCR conditions followed protocols of the Earth Microbiome Project (http://www.earthmicrobiome.org/protocols-and-standards/) (Amaral-Zettler et al., 2009, Bellemain et al., 2010, Caporaso et al., 2012). Three technical replicates were amplified for each sample, visualized with gel electrophoresis, pooled, and sent for multiplexing, library preparation, and sequencing (HiSeq 300, paired-end) at the Hubard Center for Genome Sequencing at the University of New Hampshire, Durham, NH. Qiime2 was used to remove the primer regions of the demultiplexed sequences using cutadapt (Martin, 2011). In vsearch, forward and reverse reads were joined with join-pairs (Rognes et al., 2016), joined sequences were filtered for quality with quality-filter q-score-joined, and chimeras were checked with uchime-denovo (Bokulich et al., 2013). Sequences were clustered into operational taxonomic units (OTUs) at 99% similarity. An in-house curated ARB-SILVA SSU database v.111 (Quast et al., 2012; Yilmaz et al., 2014) was used to assign taxonomy to all OTUs with BLAST. Nematode identified sequences were reassigned taxonomy against a nematode curated database based on ARB-SILVA v.138 (Gattoni et al. in review) and further processed using head–tail patterns (Porazinska et al., 2010) to generate “species-equivalent” OTUs and filtered again to retain only OTUs with a match to *Plectus*. All OTUs identified as *Plectus* were further verified with BLAST at NCBI.

### Data processing and analysis

To perform phylogenetic analyses and assess haplotype relationships among barcoded sequences of *Plectus*, traces of barcode sequences of nematode specimens were edited using CodonCode Aligner Version 9.0 (http://www.codoncode.com). Sixteen published COI and 29 18S sequences of *Plectus* were downloaded from GenBank. All DNA sequences from this study (from COI and 18S barcoding and 18S metabarcoding), NCBI, and other prairie sites were aligned with MEGAX v.10.2.6 to produce two separate (COI and 18S) alignments. Both alignments were subjected to analysis using a character-based maximum likelihood (ML) approach. ML trees were built using GTR + G + I (COI) and K2 + G + I (18S) models, both with 1,000 boot strap repetitions, and both with gap treatments using “Use all sites.” Each initial MEGAX alignment used MUSCLE with gap opening (−1,000) and gap extend (−500) and UPGMB clustering method parameters. COI haplotype groups were generally defined by the bootstrap values of ≥99, a within-group distance that did not exceed 6%, and the distance to the nearest neighbor was greater than the within-group distance. Within- and between-COI clade genetic distances were calculated in MEGAX using p-distance with the assumption of uniform rates for 124 sequences, each with 393 nucleotide positions.

## Results

### Morphological approach

**Figure 2:**
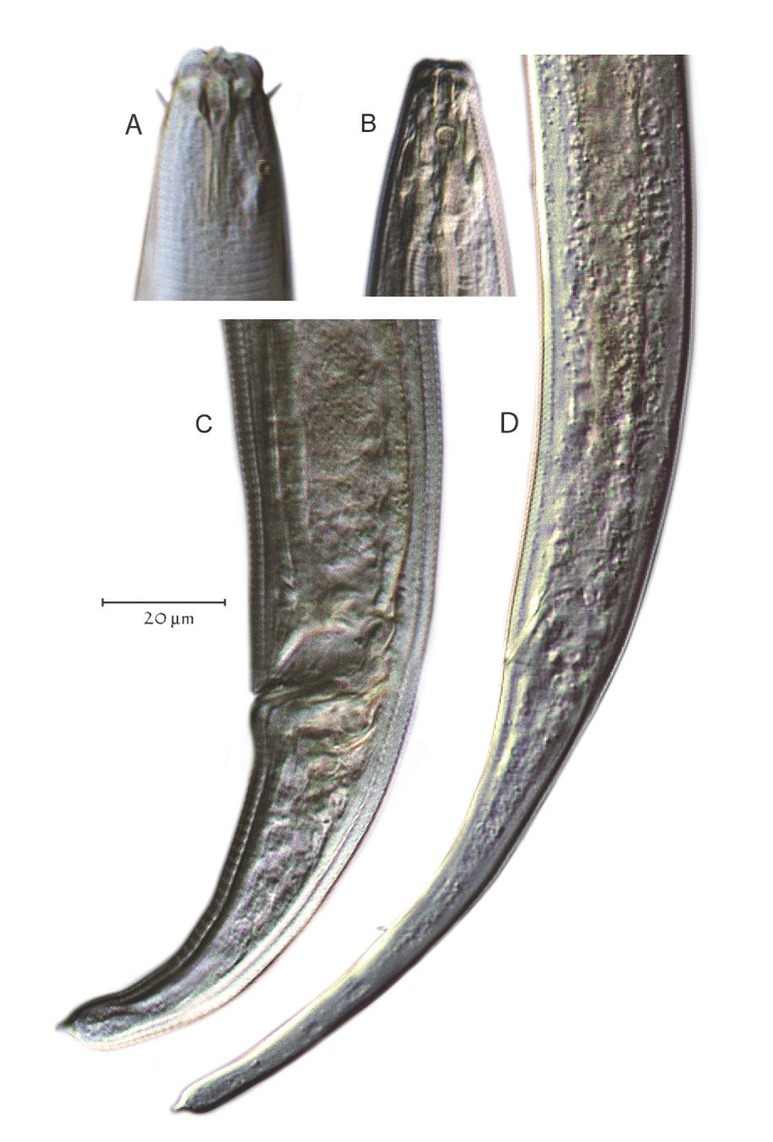
Two general *Plectus* morphotypes. (A, C) Illustrate the small amphid, short-tailed type; (B, D) Illustrate the large amphid, long-tailed type.

*Plectus* was recovered from all 15 of the remnant prairie sites investigated in this study using morphology and DNA barcoding, and from only 12 sites using metabarcoding. On an average, there were seven *Plectus* specimens per 150 nematodes at each site. In total, 101 *Plectus* corridor specimens were recorded from the sites and 44 of these were analyzed using a combination of morphological and molecular approaches. All *Plectus* observed were either females or juveniles. Of the 44 specimens that were analyzed by a combination of morphology and molecular approaches, 40% were juveniles and 60% females. Two general morphological phenotypes were observed during the microscopic sorting of corridor specimens: one with a short-tailed body, a *c´* ratio (tail length divided by anal body width) <3.0, and an amphid diameter relative to neck width ratio average of 0.14; and one with a relatively long tail with a *c´* ratio >5.0 and an amphid diameter to neck width ratio average of 0.26 (Fig. 2 and Table 2).

**Table 2:** Prairie Corridor specimen measurements from images.

Group	NID	Taxon	Stage	Initial characterization			Voucher measurements in micrometer				
				Amphid	Tail	Tail length	Anal body width	c´ ratio	Amphid width	Neck width at amphid	Amphid/ neck width (%)
3	NID	*Plectus*	F	Small,	Short	65	26.7	2.4	2	19	0.11
	12485	sp. 3		round							
3	NID	*Plectus*	J	Small,	Short	35	15	2.3	2.5	17.5	0.14
	13239	sp. 3		round							
3	NID	*Plectus*	J	Small,	Short	62	28	2.2	NA	NA	NA
	13543	sp. 3		round							
3	NID	*Plectus*	J	Small,	Short	70	29.4	2.4	2.5	NA	NA
	13030	sp. 3		round							
3	NID	*Plectus*	J	Small,	Short	61	28.9	2.1	2.5	16.6	0.15
	13045	sp. 3		round							
3	NID	*Plectus*	J	Small,	Short	65	27.5	2.4	NA	NA	NA
	13255	sp. 3		round							
3	NID	*Plectus*	J	Small,	Short	66	28.9	2.3	2	17.5	0.11
	13357	sp. 3		round							
3	NID	*Plectus*	J	Small,	Short	63	25.2	2.5	2	16.2	0.12
	13530	sp. 3		round							
3	NID	*Plectus*	J	Small,	Short	63	28.9	2.2	2	17	0.12
	13538	sp. 3		round							
3	NID	*Plectus*	J	Small,	Short	70	29	2.4	2.5	12	0.21
	11764	sp. 3		round							
3	NID	*Plectus*	J	Small,	Short	74	34	2.2	2	12	0.17
	11765	sp. 3		round							
4	NID	*Plectus*	J	Small,	Short	77	25	3.1	2.5	17	0.15
	13280	sp. 4		round							
4	NID	*Plectus*	F	Small,	Short	94	33.6	2.8	2	20.4	0.10
	13608	sp. 4		round							
6	NID	*Plectus*	J	Small,	Short	72	27.6	2.6	3	15.7	0.19
	13176	sp. 6		round							
6	NID	*Plectus*	J	Small,	Short	75	27.2	2.8	2.5	17	0.15
	13287	sp. 6		round							
6	NID	*Plectus*	J	Small,	Short	92	33.6	2.7	3	18.3	0.16
	13425	sp. 6		round							
17	NID	*Plectus*	F	Large,	Long	129	33.2	3.9	4	17.9	0.22
	13180	sp. 17		round							
17	NID	*Plectus*	F	Large,	Long	120	28.5	4.2	5	20.4	0.25
	13460	sp. 17		round							
18	NID	*Plectus*	F	Large,	Long	91.6	17.4	5.3	3.5	13.6	0.26
	13465	sp. 18		round							
19	NID	*Plectus*	F	Large,	Long	105	18	5.8	3.3	12.5	0.26
	12733	sp. 19		round							
20	NID	*Plectus*	F	Large,	Long	92.5	17	5.4	3.5	14	0.25
	13403	sp. 20		round							
20	NID	*Plectus*	F	Large,	Long	94	18.3	5.1	3.5	15.3	0.23
	13479	sp. 20		round							
20	NID	*Plectus*	F	Large,	Long	85	18.3	4.6	4	14.5	0.28
	13486	sp. 20		round							
20	NID	*Plectus*	F	Large,	Long	88	16.2	5.4	4	14.9	0.27
	13444	sp. 20		round							
20	NID	*Plectus*	J	Large,	Long	102	20	5.1	4	15.3	0.26
	13440	sp. 20		round							
20	NID	*Plectus*	F	Large,	Long	94	18.7	5.0	3	14.9	0.20
	13118	sp. 20		round							
20	NID	*Plectus*	J	Large,	Long	110	20.8	5.3	4.5	16.2	0.28
	13133	sp. 20		round							
20	NID	*Plectus*	F	Large,	Long	97	19.6	4.9	3.5	14.9	0.23
	13203	sp. 20		round							
20	NID	*Plectus*	F	Large,	Long	98	17	5.8	4.5	14.9	0.30
	13225	sp. 20		round							
20	NID	*Plectus*	F	Large,	Long	96	20.4	4.7	4	14.9	0.27
	13329	sp. 20		round							
20	NID	*Plectus*	J	Large,	Long	78	13.6	5.7	3.5	11.5	0.30
	13344	sp. 20		round							
20	NID	*Plectus*	F	Large,	Long	100	17	5.9	4	14.5	0.28
	13369	sp. 20		round							
20	NID	*Plectus*	F	Large,	Long	94	17.9	5.2	4	13.6	0.29
	13471	sp. 20		round							
20	NID	*Plectus*	F	Large,	Long	99	19.6	5.0	NA	NA	NA
	13491	sp. 20		round							
20	NID	*Plectus*	F	Large,	Long	93	19.1	4.9	3.5	15.7	0.22
	13587	sp. 20		round							
20	NID	*Plectus*	F	Large,	Long	78	17.9	4.4	4	13.6	0.29
	13598	sp. 20		round							
20	NID	*Plectus*	F	Large,	Long	91	18.3	5.0	3.5	14	0.25
	13601	sp. 20		round							
20	NID	*Plectus*	F	Large,	Long	88	16.2	5.4	4.5	14	0.32
	13622	sp. 20		round							
20	NID	*Plectus*	F	Large,	Long	108	20	5.4	3.5	14.9	0.23
	13628	sp. 20		round							
20	NID	*Plectus*	F	Large,	Long	92	18.3	5.0	3.4	14.9	0.23
	13641	sp. 20		round							

**Figure 3:**
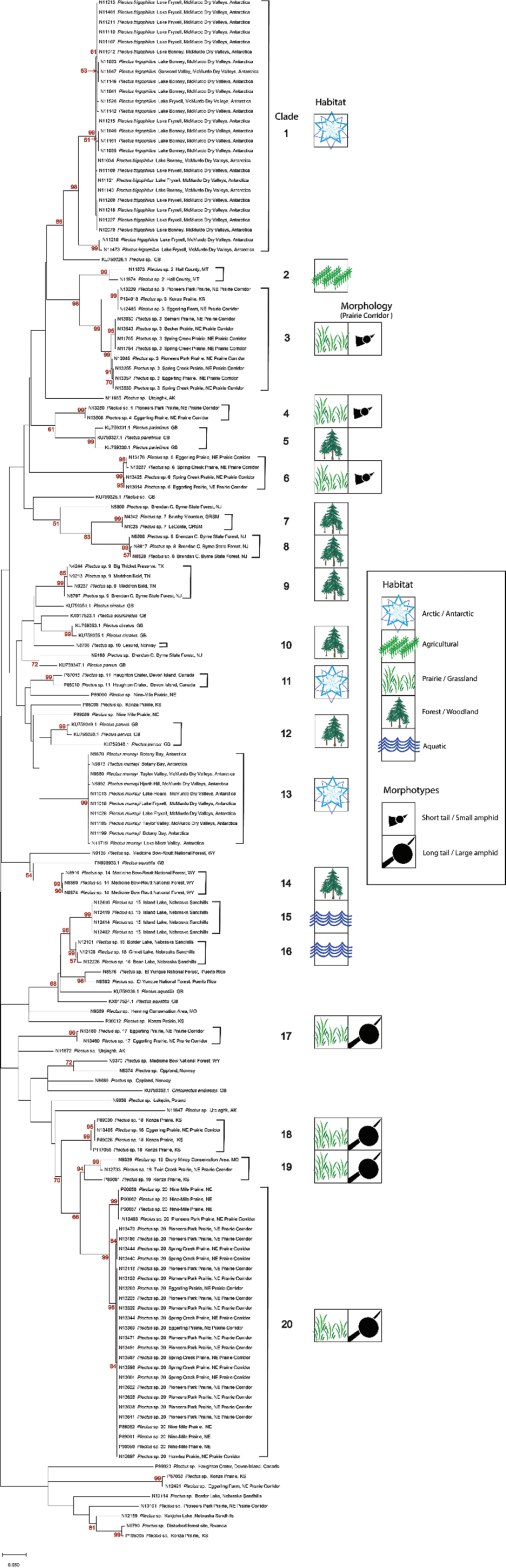
ML COI phylogenetic tree of *Plectus* specimens. Brackets denote the 20 major haplotype groups. Habitat type for each of these groups is indicated, as well as tail length-amphid size morphology for the Prairie Corridor groups. Taxon labels include the Nematode Identification Number, the genus name, and sample location. GenBank accessions are labeled with their accession number and complete name. Some haplotype groups were collapsed when identical sequences represented the group. Red numbers represent the bootstrap values from 1,000 iterations. All 44 Prairie Corridor specimens are labeled as such. ML, maximum likelihood.

**Figure 4:**
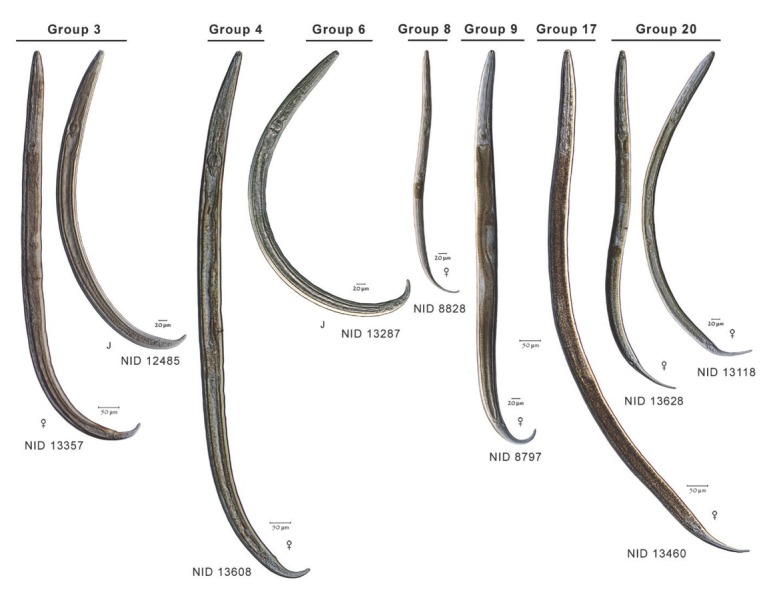
A representation of body length and overall morphology from select haplotype groups. NID and group numbers correspond to placement on the COI tree.

**Figure 5:**
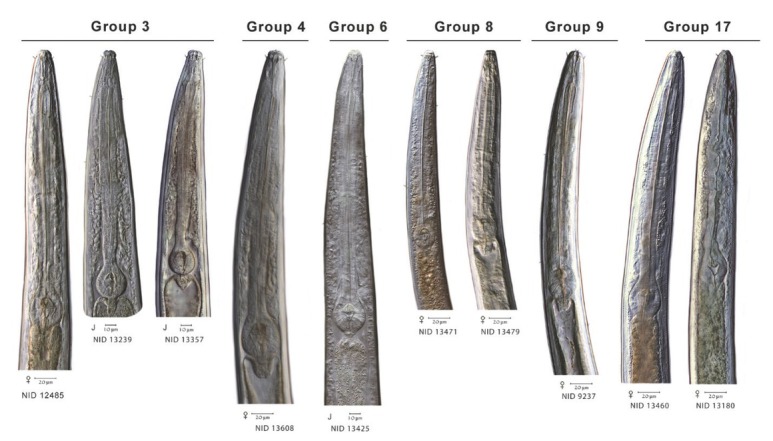
Select anterior body morphology of different haplotype groups.

**Figure 6:**
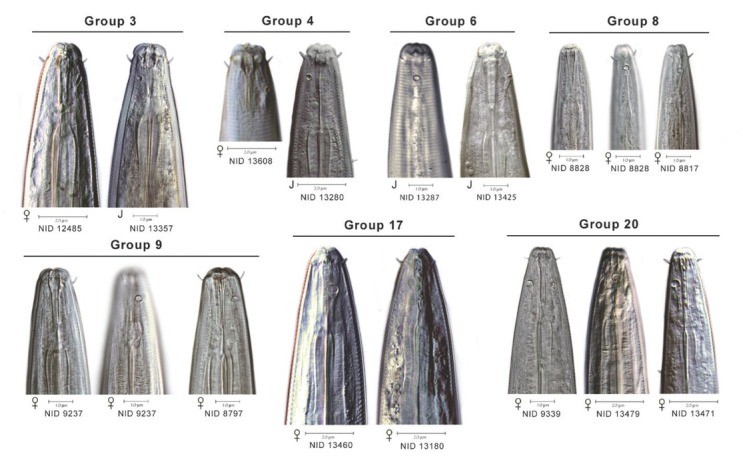
Representative cephalic regions and stomas of seven haplotype groups. Groups 3, 4, and 6 display offset cephalic regions and relatively small diameter amphids, compared to the continuous head regions and larger amphid diameters of groups 8, 9, 17, and 20. Heavier anterior sclerotization of the stoma is seen in groups 3 and 4 compared to the long, tapered stomas of 8, 9, 17, and 20.

**Figure 7:**
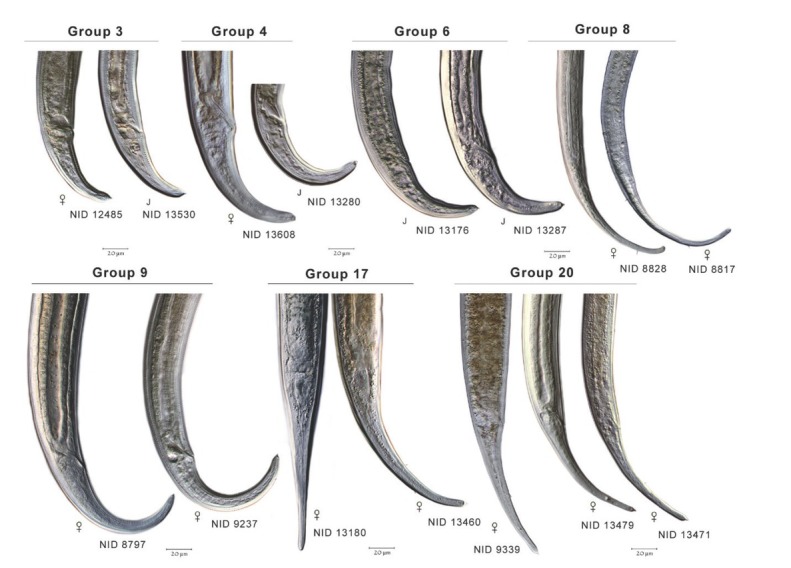
Relative tail lengths and anal body widths in different haplotype groups.

To extend comparisons beyond morphology, a second morphological assessment was conducted based on the results of DNA barcoding and phylogenetic analysis of COI sequences of voucher specimens. In this “reverse taxonomy” approach (Kanzaki et al., 2012), a phylogenetic tree of 20 clades of *Plectus*-derived COI haplotypes were recognized and subjected to further analysis (Fig. 3). Five of the 20 clades contained two or more Corridor specimens in both maximum likelihood and neighbor joining analyses. Two clades, referred to as Clades 3 and 20, contained a majority of specimens from the Corridor. Clade 3, a short-tailed morphotype, contained 10 Prairie Corridor specimens plus 1 specimen from Konza Prairie. Clade 20, a long-tailed morphotype, contained 24 Prairie Corridor specimens and six specimens from Nine-Mile Prairie. The remaining clades that contained two or more Prairie Corridor specimens were Clades 4, 6, and 17, comprised of two, four, and three specimens, respectively. Images of representative specimens from each of the corridor clades plus selected specimens from clades outside the Prairie Corridor are presented in Figures 4–7. Figure 4 illustrates a comparison of entire bodies and shows dramatic body length differences among the clades. Notable in the body length comparisons is the indication that there appear to be large and small versions of each of the two identified morphotypes. For example, the Clade 3 female in Figure 4 is 3/4ths the length of the female in Clade 4. However their *c´* ratios and amphid width to neck width ratios are very similar (Table 2). In Clade 20, the female body length is <2/3rds of the female body length in Clade 17. Again, the *c´* ratios and amphid/ neck width ratios between the females in Clades 17 and 20 are similar. Figures 5 and 6 illustrate in profile the cephalic region, stoma, and pharynx. In both figures the more heavily expanded sclerotized anterior portion of the stoma is seen in Clades 3, 4, and 6, whereas a longer, more tapered stoma is seen in Clades 17 and 20. Higher magnification in Figure 6 provides a clearer indication of amphid aperture size and amphid location on the anterior body region. The smaller amphid aperture relative to neck width is seen in Clades 3, 4, and 6. Clades 17 and 20 are characterized by larger amphid apertures relative to neck width. Figure 7 illustrates relative tail shape and length. Shorter tails relative to anal body width are evident in Clades 3, 4, and 6. Longer tails relative to anal body widths can be seen in both Clades 17 and 20. Table 2 presents measurements of tails and amphids for individual specimens in the aforementioned Prairie Corridor clades.

### DNA Barcoding approach

There were 16 COI *Plectus* accessions retrieved from GenBank that were included in the phylogenetic analyses. Additionally, 95 specimens from a University of Nebraska repository, representing collections from 1998 to 2021, were amplified and barcoded to provide additional systematic and biogeographic context for the *Plectus* COI phylogenetic tree. Two *Plectus* species from Antarctica, Clade 1 and Clade 13, as well as international specimens from Rwanda, Poland, Ireland, and Canada, were included in the phylogenetic tree. Clade 3 included one additional member from outside the corridor, a specimen from Konza Prairie in Kansas (Knapp, 1998). Clade 20 had six additional members from outside the corridor, all from 9-Mile Prairie in Nebraska. Table 3 presents the morphometrics from select non-corridor clades used for comparative purposes in the phylogenetic trees.

**Table 3:** Measurements of non-Prairie Corridor Plectus specimens.

Taxon (n)	Group 16 (3) keys to Plectus aquatilis	Group 14 (3) keys to P. cirratus	Group 9 (4) keys to P. cirratus	Group 8 (3) keys to Plectus longicaudatus	Group 13 (5) keys to P. murrayi	Group 15 (4) keys to P. palustris
L	1,394.9 ± 191.0	1,170.6 ± 78.4	1,032.7 ± 111.3	696.8 ± 35.1	883.8 ± 25.7	1,642.0 ± 141.5
	(1,183–1,646)	(1,071–1,262)	(874–1,176)	(654–740)	(840–917)	(1,411–1,778)
Tail	156.7 ± 15.3	123.8 ± 10.1	104.1 ± 10.8	100.2 ± 6.9	101.3 ± 5.2	224.7 ± 17.8
length	(145–178)	(110–135)	(88–117)	(91–107)	(94–109)	(200–246)
*a*	21.5 ± 0.7	27.3 ± 3.4	24.6 ± 2.5	26.7 ± 1.9	23.0 ± 0.8	34.2 ± 3.0
	(20.8–22.5)	(22.7–30.7)	(20.6–27.2)	(24.5–29.2)	(22.0–24.2)	(31.0–38.9)
*b*	4.5 ± 0.3	3.7 ± 0.1	4.0 ± 0.2	3.6 ± 0.0	4.1 ± 0.1	4.8 ± 0.3
	(4.2–4.9)	(3.7–3.8)	(3.6–4.1)	(3.6–3.7)	(4.0–4.2)	(4.3–5.0)
*c*	8.9 ± 0.5	9.5 ± 0.5	9.9 ± 0.1	7.0 ± 0.2	8.7 ± 0.3	7.3 ± 0.2
	(8.2–9.2)	(8.8–10.0)	(9.8–10.1)	(6.7–7.2)	(8.3–9.1)	(7.1–7.6)
*c´*	4.8 ± 0.1	5.0 ± 0.5	4.2 ± 0.3	8.1 ± 0.9	4.8 ± 0.3	8.1 ± 0.4
	(4.6–4.9)	(4.4–5.5)	(4.0–4.7)	(6.9–8.7)	(4.1–5.1)	(7.6–8.7)
V%	49.6 ± 0.9	49.8 ± 0.7	49.9 ± 0.9	47.9 ± 0.5	48.1 ± 0.4	46.2 ± 0.5
	(48.4–50.5)	(48.8–50.6)	(49.0–51.3)	(47.2–48.5)	(47.8–48.9)	(45.7–47.0)
V–A/T	3.5 ± 0.3	3.8 ± 0.3	4.0 ± 0.1	2.6 ± 0.1	3.5 ± 0.2	2.9 ± 0.1
	(3.1–3.8)	(3.4–4.1)	(3.8–4.1)	(2.6–2.8)	(3.3–3.8)	(2.8–3.1)
LR W/H	2.5 ± 0.3	2.6 ± 0.3	2.7 ± 0.1	2.0 ± 0.4	2.3 ± 0.3	2.0 ± 0.2
	(2.0–2.8)	(2.3–3.0)	(2.6–2.8)	(1.5–2.3)	(2.1–2.7)	(1.6–2.2)
rec/abw	0.8 ± 0.1	1.2 ± 0.1	1.0 ± 0.2	1.9 ±0.2	1.0 ± 0.2	1.2 ± 0.1
	(0.7–0.9)	(1.1–1.3)	(0.8–1.3)	(1.7–2.2)	(0.7–1.1)	(1.1–1.3)
Ceph	3.5 ± 0.6 (3–4)	3.5 ± 0.9 (3–5)	3.7 ± 0.8 (3–5)	3.2 ± 0.1 (3–3)	3.4 ± 0.5 (3–4)	3.0 ± 0.2 (3–3)
set L						
Ceph	5.9 ± 0.6 (5–7)	5.8 ± 0.8 (5–7)	6.5 ± 1.0 (5–8)	5.1 ± 0.5 (4–6)	5.5 ± 0.8 (5–7)	6.0 ± 1.0 (5–7)
set pos						
Set L/	26.5 ± 5.5	25.5 ± 3.1	29.1 ± 5.3	38.7 ± 9.4	31.3 ± 6.1	24.5 ± 1.9
lrw%	(19.3–32.7)	(22.3–29.7)	(22.4–35.2)	(31.6–52.0)	(21.2–39.5)	(22.9–27.7)
Amphid	3.5 ± 0.4 (3–4)	3.3 ± 0.2 (3–3)	3.5 ± 0.5 (3–4)	3.1 ± 0.1 (3–3)	2.5 ± 0.2 (2–3)	3.4 ± 0.3 (3–4)
W						
Amp	0.9 ± 0.1	0.9 ± 0.0	0.8 ± 0.1	0.7 ± 0.1	1.0 ± 0.1	1.0 ± 0.2
L/W	(0.8–1.0)	(0.9–0.9)	(0.6–0.9)	(0.6–0.9)	(0.9–1.2)	(0.8–1.3)
Amphid	12.3 ± 0.5	10.3 ± 1.4	13.0 ± 1.1	8.4 ± 0.6 (8–9)	11.2 ± 1.1	14.2 ± 0.5
pos	(12–13)	(8–12)	(12–15)		(10–13)	(13–15)
Amp W/	19.9 ± 2.8	18.6 ± 2.5	19.8 ± 2.8	26.6 ± 0.9	15.5 ± 2.0	18.5 ± 2.1
head%	(17.5–23.9)	(15.2–20.9)	(15.8–23.3)	(25.5–27.7)	(13.2–18.5)	(16.0–21.7)
Stoma L	24.3 ± 0.1	24.3 ± 3.5	24.1 ± 4.3	18.3 ± 2.2	21.4 ± 3.8	27.0 ± 2.1
	(24–24)	(19–28)	(20–31)	(17–21)	(16–27)	(24–30)
Stoma	7.4 ±1.2	6.7 ± 2.0	7.3 ± 2.1	5.5 ± 0.7	8.7 ± 3.5	9.5 ± 1.3
L/W	(6.1–8.9)	(4.6–9.5)	(4.2–9.5)	(4.8–6.2)	(6.0–15.4)	(7.5–10.9)
Sto W/	21.8 ±1.8	26.7 ± 7.8	24.6 ±7.3	31.9 ± 2.2	21.3 ± 4.8	20.8 ± 2.1
head%	(19.3–23.5)	(16.2–34.7)	(19.7–37.2)	(29.7–34.1)	(12.3–26.7)	(18.3–24.1)
Prosto/	30.3 ± 2.0	24.8 ± 7.0	32.1 ± 4.1	31.2 ± 1.0	22.8 ± 4.4	30.8 ± 2.7
sto%	(27.8–32.7)	(18.3–34.6)	(27.8–38.8)	(29.9–32.4)	(15.7–28.6)	(27.8–35.1)
Exc	56.3 ± 0.8	54.1 ± 0.8	53.9 ± 3.5	54.1 ± 1.6	57.2 ± 0.7	56.2 ± 0.7
Pore%	(55.6–57.4)	(53.1–55.1)	(48.3–57.1)	(52.5–55.7)	(56.4–58.5)	(55.4–57.0)
Cerv	208.9 ± 50.2	192.1 ± 27.3	147.2 ± 13.6	90.0 ± 0.0	130.9 ± 3.2	202.3 ± 8.3
papilla	(167–279)	(165–229)	(134–170)	(90–90)	(126–134)	(191–214)
Body	1.7 ± 0.3	1.2 ± 0.0	1.6 ± 0.1	1.0 ± 0.0	1.3 ± 0.1	1.5 ± 0.2
ann W	(1.3–2.0)	(1.1–1.2)	(1.4–1.7)	(0.9–1.1)	(1.2–1.4)	(1.2–1.7)
G’1%	25.0 ± 1.7	19.6 ± 2.9	20.5 ± 2.7	14.0 ± 0.7	16.6 ± 2.2	20.0 ± 2.0
	(23.3–26.7)	(15.9–23.0)	(16.5–23.3)	(12.9–14.6)	(13.5–19.8)	(17.9–22.4)
G’2%	25.5 ± 2.8	18.7 ± 3.1	18.2 ± 3.2	17.1 ± 2.6	18.0 ± 3.4	20.1 ± 2.0
	(22.7–28.2)	(14.7–22.4)	(13.4–21.3)	(14.3–20.5)	(14.4–22.3)	(17.0–22.2)
Spin	1.5 ± 0.1	1.7 ± 0.2	1.2 ± 0.1	1.1 ± 0.5	1.3 ± 0.4	2.0 ± 0.6
L/W	(1.3–1.6)	(1.5–2.0)	(1.1–1.4)	(0.6–1.6)	(0.8–1.9)	(1.3–2.8)

The seven clades with the Prairie Corridor species (Clades 3, 4, 6, 17, 18, 19, and 20) were exclusively comprised of specimens collected from native prairie habitats. *Plectus* specimens from the New Jersey Pine Barrens (Clade 8), Great Smoky Mountains (Clade 7), Medicine Bow National Forest in Wyoming (Clade 14), Devon Island, Canada (Clade 11), the Antarctic Dry Valleys (Clades 1, 13), and the Alkaline Lakes in the western Sandhills of Nebraska (Clades 15, 16) all exhibited location-specific *Plectus* taxa. Only a single clade, Clade 9, had well-supported, closely related sequences collected from geographically distant sites. This clade included a mountaintop heath bald in the Great Smoky Mountains, the New Jersey Pine Barrens, and Big Thicket National Preserve in Texas.

Genetic distances within and between the 20 COI Clades are presented in Table 4. Although sample sizes varied, the two Antarctic clades were notable for low levels of within-group polymorphism. Between-group mean pairwise genetic distance varied from 0.0458 to 0.1806 for the 20 clades. The lower value represents mean genetic distance between specimens from different lakes in the western Nebraska Sandhills that were initially separated due to non-overlapping morphological characteristics (Table 3). The highest distance value is between specimens from a wheat field in Montana and Clade 19, a geographically heterogeneous group of grassland specimens.

**Table 4:** Within-^a^ and between-group distances.

	Plectus frigophilus	Plectus sp. 2	Plectus sp. 3	Plectus sp. 4	Plectus sp. 6	Plectus sp. 7	Plectus sp. 8	Plectus sp. 9	Plectus sp. 10	Plectus sp. 11
*Plectus frigophilus*	**0.01445**									
*Plectus* sp. 2	0.145469	**0.0458**								
*Plectus* sp. 3	0.132330	0.105149	**0.02642**							
*Plectus* sp. 4	0.144087	0.152672	0.153299	**0.00254**						
*Plectus* sp. 6	0.164902	0.144402	0.142419	0.136768	**0.00636**					
*Plectus* sp. 7	0.161954	0.152672	0.162706	0.153944	0.169847	**0**				
*Plectus* sp. 8	0.147804	0.152248	0.148707	0.148007	0.155428	0.094996	**0.00339**			
*Plectus* sp. 9	0.158646	0.164758	0.172045	0.123410	0.152672	0.133588	0.147371	**0.00763**		
*Plectus* sp. 10	0.146456	0.148855	0.149938	0.115776	0.157125	0.124682	0.118745	0.086514	**n/c^b^**	
*Plectus* sp. 11	0.146744	0.150763	0.146703	0.131043	0.163486	0.141221	0.154368	0.113868	0.124682	**0.03053**
*Plectus murrayi*	0.147902	0.166545	0.155100	0.138186	0.175413	0.156327	0.163139	0.100769	0.123120	0.100899
*Plectus* sp. 14	0.142599	0.149703	0.147779	0.139525	0.155428	0.141645	0.139949	0.118533	0.106022	0.114080
*Plectus* sp. 15	0.173392	0.161578	0.172686	0.151399	0.174936	0.145038	0.153520	0.128499	0.114504	0.133588
*Plectus* sp. 16	0.169633	0.170059	0.168288	0.152248	0.166455	0.143342	0.146735	0.132740	0.116200	0.120865
*Plectus* sp. 17	0.165902	0.176845	0.164923	0.137405	0.144402	0.153944	0.172604	0.145038	0.138677	0.131679
*Plectus* sp. 18	0.163522	0.174936	0.170885	0.146310	0.157761	0.152672	0.158609	0.148855	0.131679	0.132316
*Plectus* sp. 19	0.176055	0.180662	0.169356	0.152248	0.160517	0.146735	0.153520	0.151612	0.134012	0.133164
*Plectus* sp. 20	0.175147	0.172392	0.166065	0.148219	0.160760	0.156398	0.165243	0.157397	0.130225	0.128862
*Plectus* sp. 5	0.130518	0.148551	0.143198	0.107337	0.163043	0.163043	0.150060	0.156703	0.142210	0.151721
*Plectus* sp. 12	0.147765	0.161232	0.156868	0.132699	0.161232	0.154891	0.162138	0.110960	0.134964	0.101449

	Plectus murrayi	Plectus sp. 14	Plectus sp. 15	Plectus sp. 16	Plectus sp. 17	Plectus sp. 18	Plectus sp. 19	Plectus sp. 20	Plectus sp. 5	Plectus sp. 12
*Plectus* sp. 13	0.0012									
*Plectus* sp. 14	0.120908	0.00339								
*Plectus* sp. 15	0.114939	0.118745	0							
*Plectus* sp. 16	0.122602	0.121289	0.045802	0.00679						
*Plectus* sp. 17	0.141001	0.138253	0.146310	0.148855	0.01018					
*Plectus* sp. 18	0.145731	0.144614	0.136768	0.118957	0.136768	0.00382				
*Plectus* sp. 19	0.134193	0.133164	0.147583	0.137970	0.124258	0.108355	0.05513			
*Plectus* sp. 20	0.146096	0.131376	0.134224	0.128287	0.138041	0.088877	0.088422	0.00837		
*Plectus* sp. 5	0.137681	0.147645	0.132246	0.137379	0.164855	0.157835	0.170290	0.169999	0.01993	
*Plectus* sp. 12	0.100543	0.121679	0.123188	0.128623	0.132246	0.143569	0.136775	0.142048	0.149457	0.06612

a within-group distance values in bold. ^b^Not able to be calculated.

**Figure 8:**
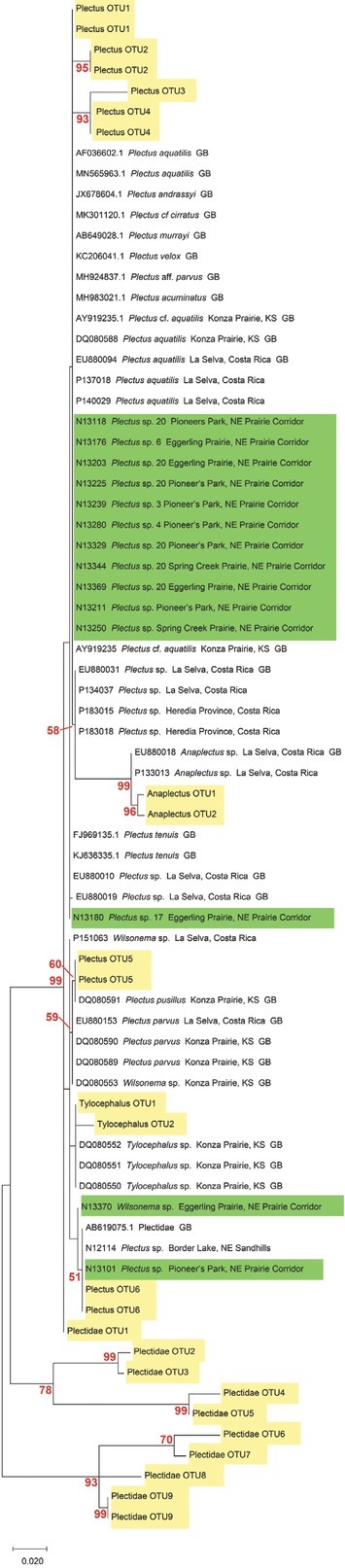
ML tree of 18S sequences. OTUs from metabarcoding are highlighted in yellow. Sanger sequences from the prairie corridor are highlighted in green. Numbers following the genus name correspond to COI clade. GenBank accessions are included with their full species name. ML, maximum likelihood; OTUs, operational taxonomic units.

Figure 8 displays the 18S tree with individual barcodes generated by Sanger sequencing, GenBank accessions, and sequences generated by metabarcoding. Sequences from split template specimens that were also barcoded with COI are identified by their Clade number following their genus name as designated on the COI phylogenetic tree. There was little nucleotide sequence differentiation among Prairie Corridor *Plectus* specimens for the 18S NF1 region, with only two specimens (NID 13101 and NID 13180) exhibiting sequence variation when compared to other Prairie Corridor specimens. The COI barcoded specimens of Clades 2, 3, 6, 17, and 20 all produced identical 18S sequence for the NF1 region (Fig. 8). GenBank accessions of seven different named species also exhibited identical 18S sequence in the NF1 region.

### Metabarcoding approach

There were 19 OTUs representing Plectida produced by the NF1 metabarcoding analysis. Not all of these could be classified as members of the genus *Plectus*, but could be assigned to a higher taxon within the order Plectida. *Plectus* OTU1 matched the 18S sequence that characterized the majority of specimens sequenced by Sanger sequencing and 12 GenBank accessions. OTU 6 matched N13101, a *Plectus* singleton on the COI tree. Other OTU sequences matched different plectid genera such as *Anaplectus* and *Tylocephalus*, in addition to other unidentified taxa.

## Discussion

The North American Prairies are known for their rich plant and animal diversity (Savage, 2011). Surveys of prairie nematodes have supported the idea of high biotic diversity (Orr and Dickerson, 1966; Todd et al., 2006). Since the initial, historical nematode surveys were conducted using traditional morphological approaches as visualized by light microscopy, it could be expected that molecular approaches will provide equivalent, or even higher, diversity estimates. This expectation was met, although determination of specimen species identity in the seven prairie clades and additional prairie singletons on the COI tree was difficult.

We selected *Plectus* as a case study of nematode diversity as revealed by different assessment methods, because of its frequent occurrence within the 15 remnant prairie sites in the Lancaster County Prairie Corridor. Orr and Dickerson (1966) identified seven *Plectus* species from tallgrass prairie in Kansas and applied Latin binomials for six of them. Most of these species were initially described from England or Europe, in the second half of the 19th century. Their presence in the native tallgrass prairies of North America may reflect the resolution of early morphological keys rather than an example of widespread dispersal of a microbial-feeding nematode. Our initial microscopic examination recognized two *Plectus* morphotypes primarily differentiated by their tail length and the size of their amphid apertures. Further taxonomic discrimination by morphology was hampered by the high percentage of juveniles in these sites (in the case of Clade 6, only juveniles were observed) and the limited amount of morphological information we could obtain from photographic vouchers. Therefore, we employed reverse taxonomy, an approach where the phylogenetic trees define the taxonomic units and provide the structure in which to investigate taxonomic identity of groups in greater detail. This approach sorted the Prairie Corridor specimens iinto seven groups among 20 total groups of *Plectus* specimens representing multiple habitats and locations. Over 70% of the 45 barcode-analyzed *Plectus* specimens from the Prairie Corridor fell into two clades, Clades 3 and 20. The taxonomic identities of these groups, however, were not matched by reference sequences in GenBank. There are six GenBank species of *Plectus* with Linnaean binomials on the COI phylogenetic tree, and three of them are located in multiple clades. The greatest similarity between a GenBank specimen and a Prairie Corridor specimen is found in Clade 4, which is in a sister group relationship with Clade 5; and the latter is represented by three *Plectus parietinus* (KU759327, KU759330, and KU759331). These specimens were collected from moss in a German beech forest (Schenk et al., 2016) and exhibit a weak bootstrap support value of 61 and a pairwise group dissimilarity of 10.7% with Clade 4. Could the Prairie Corridor Clade 4 actually be *P. parietinus?*

**Figure 9:**
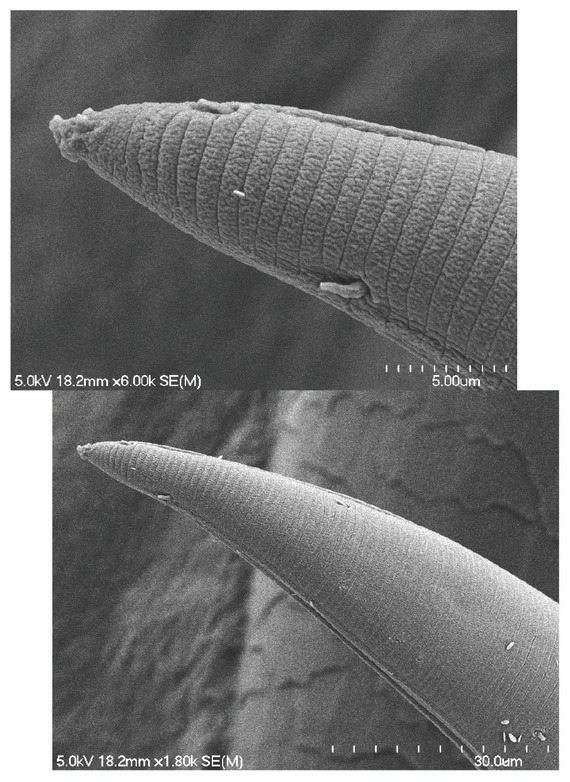
Scanning electron micrograph of short-tailed specimen exhibiting caudal setae near tail terminus on dorsal surface.

Morphologically, Prairie Corridor specimens in Clades 3, 4, and 6 resemble *P. parietinus*. The dichotomous key and notes of Maggenti (1961) lead to a *P. parietinus* designation based on the set-off lip region, a tail length less than six anal body diameters, an amphid diameter of 2.5 mm, and a subdorsal seta near the tail tip (Fig. 9). The *c´* values in the keys of Andrássy (1985), Bongers (1989), and Ebsary (1985) specify values of 3 to 4, whereas the corresponding Prairie Corridor specimen *c´* values were smaller, with ratios of 2 to 3. The key of Andrássy also emphasizes the strongly sclerotized anterior part of the stoma as characteristic of *P. parietinus*. A comparison of the mean COI pairwise dissimilarity values of Clades 3, 4, and 6 indicates that these are between 13.7% and 15.3%. These dissimilarity values are roughly equivalent to many pairwise comparisons across the 20-clade COI tree, suggesting that genetic distance alone is insufficient for distinguishing among these clades. Given the information, at best we can say that members of these three clades morphologically resemble *P. parietinus*, but do not constitute an exact match morphologically or genetically.

**Figure 10:**
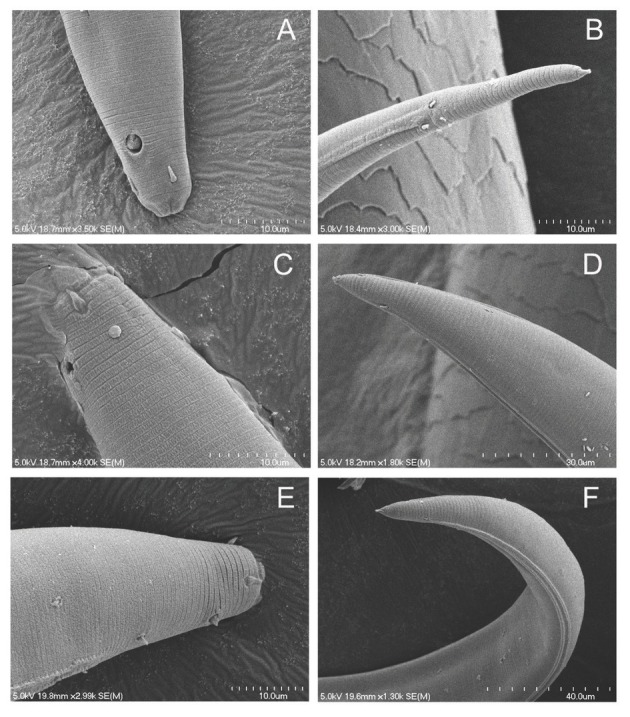
Scanning Electron Micrograph of corresponding heads and tails of long-tailed morphotype (A, B) and short-tailed morphotypes (C, D and E, F).

The most common *Plectus* haplotype group in the Prairie Corridor was Clade 20. Clade 20 is represented by 28 specimens from the Prairie Corridor and Nine-Mile Prairie. Prairie Corridor Clades 17, 18, 19, and 20 belong to the long-tailed, large amphid morphotype (Fig. 10). There are no COI sequences that are closely related to Clade 20 in GenBank. Morphologically, keys lead to an identification of *P. rhizophilus* based on a lip region that is not set-off, a *c´* measurement of >4, an amphid diameter that is approximately one quarter of the neck width, and cephalic setae that do not reach the apex of the lip region. Body length of Clade 20 is within the range for *P. rhizophilus*, but specimens in Clade 17 exceed the reported length for that species.

Species identification for specimens outside the Prairie Corridor could be considered educated guesses at best. There are two clades that are distinctive based on morphology and location. The two Antarctic clades represent well-studied, homogeneous species known only from specific habitats on the Antarctic continent (Kito et al., 1991). *Plectus frigophilus* Kirjanova, 1958, (Clade 1) inhabits the sediment of glacial lakes whereas *Plectus murrayi* (Clade 13) is most often found on moss and cyanobacterial mats along glacial melt streams. Another non-corridor clade that is distinctive in this dataset is Clade 8, which is characterized by a relatively short body length (<900 mm) and a long, slender tail with *c´* value >8. The specimens key and conform in form to *P. longicaudatus* Butschli, 1873, with the exception that the tail is not curved or twisted in the distal third portion and the body length exceeds the measurements of Yeates (1988) and Andrássy (1985). In general, North American species may have some morphological similarities with described European taxa, but without direct sequence comparisons with unimpeachably identified reference specimens, we cannot be certain of their species identity. Therefore, until more detailed studies are conducted on individual species, in our opinion it is best to designate these taxonomically uncertain species as *Plectus* sp. followed by their clade number.

The uniformity in 18S sequence in the NF1 region indicates that neither DNA barcoding nor metabarcoding analysis based on this portion of 18S alone will allow discrimination among *Plectus* species of the Prairie Corridor. The inability of 18S to discriminate among *Plectus* species has been noted by other authors (Shokoohi et al., 2013; Schenk et al., 2016). The limitation of 18S in metabarcoding assessments of diversity, which will bias measurements toward greater uniformity, could be countered by the development of a metabarcording approach incorporating mitochondrial genes.

COI haplotype groups may provide insight into *Plectus* phylogeography. There appears to be a fairly high level of regional localization within the *Plectus* COI clades and negligible evidence for long distance dispersal or cosmopolitan distribution. This is unexpected because *Plectus* is a bacterial feeder and presumably can feed on a range of bacteria. Given that many species are parthenogenetic and may readily enter an anhydrobiotic state, observation of geographically widespread identical COI haplotypes might be expected. We did not observe this. There may be physiological or ecological factors that structure *Plectus* populations. In this study, most of the sampled nematodes were from native plant communities and unlikely to have been affected by anthropogenic disturbances associated with cultivated soils. Agricultural soils, however, are well-known to harbor plant-parasitic nematodes with cosmopolitan distributions. Future studies using a taxonomic marker like COI coupled to detailed morphological and ecological analyses, should lead to a greater understanding of *Plectus* diversity and the factors that structure their populations.
